# Reality of Osteoporosis in Primary Healthcare: An Observational Study in a Portuguese Local Health Unit

**DOI:** 10.7759/cureus.100582

**Published:** 2026-01-01

**Authors:** Nuno Pinto, Ana Machado, Ana Matos, Cláudia S Cardoso, João Martins Figueiredo, João Carlos Silveira, Joao De Oliveira e Silva, Diana Gonçalves, Paula Valente, Maria Helena Lourenço, Rita Fonseca

**Affiliations:** 1 Family Medicine, Unidade Local de Saúde Entre Douro e Vouga, Santa Maria da Feira, PRT; 2 Rheumatology, Unidade Local de Saúde da Póvoa de Varzim/Vila do Conde, Póvoa de Varzim, PRT; 3 Rheumatology, Unidade Local de Saúde Entre Douro e Vouga, Santa Maria da Feira, PRT

**Keywords:** bisphosphonates, drug holiday, dual-energy x-ray absorptiometry (dxa), fracture risk assessment tool, frax, osteoporosis, osteoporotic fracture, primary healthcare

## Abstract

Introduction: Osteoporosis (OP) is a common chronic condition associated with an increasing burden of fragility fractures and healthcare costs. Risk assessment and management rely on tools such as the Fracture Risk Assessment Tool (FRAX®) and dual-energy X-ray absorptiometry (DXA), yet their application in routine primary healthcare (PHC) practice remains unclear.

Objectives: To characterize real-world OP screening and management practices in a PHC setting.

Materials and methods: A single observational study with two predefined cohorts was conducted across five family health units (FHUs). The screening cohort included individuals aged exactly 50 years, women aged exactly 65 years, and men aged exactly 70 years attending consultations at those ages. The OP cohort included patients with a confirmed diagnosis of OP established between 2013 and 2023. Data were collected from electronic medical records, anonymized, and analyzed using descriptive statistical methods (IBM^®^ SPSS^®^ Statistics 25.0 (IBM Corp., Armonk, NY, USA)). Ethical approval was obtained from the Ethics Committee of the Unidade Local de Saúde Entre Douro e Vouga, Santa Maria da Feira, Portugal, which oversees all participating FHUs.

Results: In the screening cohort (n=770), FRAX® was not registered in 99.6% of individuals aged 50 years. DXA was not requested in 87% of women aged 65 years and 97.2% of men aged 70 years. In the OP cohort (n=679), 49.2% of patients with a diagnosis duration of two years or more underwent follow-up DXA. Physician-advised bisphosphonate discontinuation occurred in 14.4% of cases and was considered appropriate in 61.5%, based on predefined clinical criteria. In contrast, most patient-initiated bisphosphonate discontinuations occurred without documented clinical justification. Although 16.9% of patients met criteria for referral (bisphosphonate refractoriness, osteoporotic fracture, or suspected secondary OP), 75.7% were not referred to rheumatology.

Conclusion: OP screening and management in primary healthcare showed low utilization of FRAX® and DXA, inconsistent follow-up, suboptimal long-term treatment adherence, and limited referral to rheumatology, reflecting gaps in real-world clinical practice.

## Introduction

Osteoporosis (OP) is a metabolic bone disorder characterized by compromised bone strength and low bone mineral density (BMD), predisposing individuals to fragility fractures [1]. Epidemiological data in Portugal indicate that approximately 10.2% of the adult population self-report having OP [2], while around 5.6% meet diagnostic criteria based on BMD, a prevalence similar to the European Union (EU) average [3,4]. Although mortality rates in Portugal due to OP-related events are lower than the EU mean, the incidence of fragility fractures and the economic burden of OP-related complications have been increasing since 2010, with higher values expected by 2034 [4,5].

Although safe, efficacious, and economical medications are available, OP is underdiagnosed and often untreated; its screening and treatment should be part of regular practice in the primary healthcare (PHC) setting. Healthcare professionals should consider assessing this risk for patients ≥ 40 years old, using the Fracture Risk Assessment Tool (FRAX®), which is a validated tool for deciding whether a dual-energy X-ray absorptiometry (DXA) scan and/or treatment is necessary [6,7]. Another way to detect OP early is through DXA, which is indicated in women aged 65 years or older and men aged 70 years or older [8].

Guidelines regarding follow-up vary across international societies, with recommended intervals for DXA monitoring ranging from one to five years after the initiation of anti-osteoporotic therapy. In Portugal, the most common practice is to perform DXA re-evaluation at intervals of no less than two years [9-11].

Bisphosphonates are considered the first line of therapy for OP in several countries. Although generally well-tolerated, there are some concerns about their long-term use, including atypical femur fractures and osteonecrosis of the jaw. Therefore, a temporary interruption of bisphosphonate therapy, known as a "drug holiday" (DH), may be reasonable to minimize these risks. In Portugal, recommendations suggest suspending bisphosphonate use for two to three years if the following conditions are cumulatively met: no new fragility fractures observed during treatment, adherence to therapy for five years, and a femoral neck T-score greater than −2.5 [5,10].

Ultimately, referral to the rheumatology department may be necessary. These criteria vary across hospitals but generally include osteoporotic fracture (with or without previous therapy), suspicion of secondary OP, adverse effects related to therapy, and doubts regarding diagnosis or treatment [12].

In light of the above, this study aims to investigate the Portuguese reality in the PHC of diagnosis and management of OP. The primary goals included understanding if FRAX® is being carried out in patients aged 50 years and if DXA is being requested at key ages, especially in women aged 65 years and men aged 70 years, as well as regularly during the follow-up period. Moreover, the authors aim to clarify if bisphosphonates are being suspended whenever criteria are met and if referrals to the rheumatology department are being carried out in accordance with the established criteria.

## Materials and methods

Our study employed a single observational design with two complementary methodological components, aimed at capturing different dimensions of real-world OP care in PHC. Specifically, the study comprised a cross-sectional assessment of OP screening practices conducted in 2023 and a retrospective analysis of OP management and follow-up between 2013 and 2023. This combined approach was intentionally chosen to allow the evaluation of current screening practices at predefined guideline-recommended age thresholds, while also enabling the assessment of long-term follow-up, pharmacological management, treatment discontinuation, and referral patterns in patients with established OP.

Participants were recruited from five family health units (FHU)-Unidade de Saúde Familiar (USF) Aliança, USF Nordeste, USF Salvador Machado, USF São João, and USF Vale do Vouga-within the PHC setting of the Portuguese regions of São João da Madeira and Oliveira de Azeméis, all operating under the Local Health Unit (ULS) Entre Douro e Vouga in Santa Maria da Feira, Portugal.

The study population was divided into two predefined cohorts within the same observational framework: a screening cohort and an OP cohort. Distinct eligibility criteria were applied to each cohort. In the screening cohort, we included individuals without a prior diagnosis of OP who attended consultations at exactly predefined ages in 2023: individuals aged exactly 50 years, women aged exactly 65 years, and men aged exactly 70 years. These fixed age thresholds were selected because they correspond to key preventive milestones and guideline-recommended ages for OP screening in routine clinical practice. In particular, the 50-year cut-off represents a stage at which several preventive and screening interventions are routinely initiated in PHC, allowing the assessment of whether OP risk evaluation is incorporated into consultations already focused on preventive care. In the OP cohort, we included patients with a confirmed diagnosis of OP established between 2013 and 2023. For both cohorts, exclusion criteria comprised pregnant women, deceased patients, and individuals already under hospital-based follow-up. Additionally, patients with misclassified diagnoses, such as osteopenia erroneously coded as OP, were excluded from the OP cohort.

Participant identification was performed using the Módulo de Informação e Monitorização das Unidades Funcionais (MiM@UF). Clinical data were extracted from electronic medical records using SClínico®, while the Registo de Saúde Eletrónico (RSE) was used to verify exclusion criteria related to hospital-level care. Past pharmacological prescriptions were reviewed through the Prescrição Eletrónica de Medicamentos (PEM) platform. These electronic systems are uniformly implemented across all participating FHUs, allowing centralized data collection across sites.

For the screening cohort, collected variables included sex and age, application of FRAX® in individuals aged exactly 50 years, and referral for DXA in women aged exactly 65 years and men aged exactly 70 years. Data corresponded to consultations occurring at those exact ages during 2023. Information on prior DXA referrals and their appropriateness was also retrieved from historical medical and prescription records.

For the OP cohort, collected variables included pharmacological treatment, tolerability to bisphosphonate therapy, occurrence of fractures during treatment, adherence to bisphosphonate therapy for a minimum duration of five years, DXA requests during follow-up, DXA requests after five years of bisphosphonate therapy, bisphosphonate suspension and its justification, and referral to the rheumatology department according to predefined clinical criteria. Tolerability was assessed based on documentation of adverse effects in electronic medical records and related clinical decisions, such as treatment discontinuation or modification; in the absence of documented adverse effects, treatment was considered tolerated. Five-year adherence was assessed through continuous electronic prescription records in the PEM platform, reflecting treatment persistence over that period. DXA requests after five years of bisphosphonate therapy were initiated by the PHC physician as part of routine follow-up, with the purpose of reassessing BMD and supporting decisions regarding treatment continuation, modification, or suspension.

DH criteria were explicitly defined as the cumulative presence of the following conditions: absence of new fragility fractures during bisphosphonate therapy, documented adherence to treatment for at least five years, and a femoral neck T-score greater than −2.5 on follow-up DXA. Suspension of bisphosphonates was categorized according to the documented reason, including fulfillment of DH criteria, occurrence of adverse effects, lack of therapeutic efficacy (such as fractures or absence of improvement in BMD), and patient-initiated discontinuation without medical recommendation. Referral to the Rheumatology department was assessed according to predefined clinical criteria, including bisphosphonate refractoriness (fractures during therapy or lack of improvement in BMD on follow-up DXA), osteoporotic fractures at diagnosis or during follow-up, suspected secondary OP, and uncertainty regarding diagnosis or treatment.

The numerical variables assessed included the time interval, in years, between the initial OP diagnosis and follow-up DXA assessment, and the femoral neck T-score obtained from the DXA performed after five years of bisphosphonate therapy, which was used to support clinical decision-making.

All data were anonymized and stored securely. Statistical analysis was performed using descriptive statistical methods, with categorical variables summarized as frequencies and percentages, and continuous variables presented as means and medians to account for potential non-normal distributions. Analyses were conducted using IBM® SPSS® Statistics 25.0 (IBM Corp., Armonk, NY, USA).

Ethical approval was granted by the Ethics Committee of the ULS Entre Douro e Vouga on 13th June 2024 (n.º 24_2024). This committee provides ethical oversight for all participating FHUs, and the approval covered the entire observational study protocol, including both the cross-sectional and retrospective components.

## Results

Screening cohort

FRAX® Application

Regarding FRAX® application to the population aged 50 years, 778 patients from the study-enrolled FHUs in 2023 were included. Seven patients were then excluded due to a pre-existing OP diagnosis, and a further one due to concurrent management by hospital-based appointments. After applying the exclusion criteria, 770 individuals aged exactly 50 years were included in the screening cohort component assessing FRAX® application. For the majority of these patients (99.6% (n=767)) FRAX® was not applied by the PHC physician. Among the three patients assessed by FRAX® tool, the mean major osteoporotic fracture risk was 1.6%, and the mean hip fracture risk was 0.2%. 

DXA Prescription

Concerning the DXA prescription component, focused on women aged 65 years and men aged 70 years, the researchers identified 839 patients who attended appointments in 2023. Of these, 35 women and one man were excluded due to a prior diagnosis of OP. Following the application of exclusion criteria, 477 women aged exactly 65 years and 326 men aged exactly 70 years were included. As for women aged 65 years, DXA was not prescribed during any of their 2023 appointments in 87% of cases (n=415). Furthermore, in 13.2% (n=63) of these women, a prior DXA had been conducted, but in 57.1% of these cases, clinical criteria were lacking. Regarding men, DXA was similarly absent during all appointments in 2023 in 97.2% (n=317) of cases. Additionally, 3.4% of these men (n=11) had previously undergone DXA, without clinical criteria in 63.6% of these situations. 

**Figure 1:**
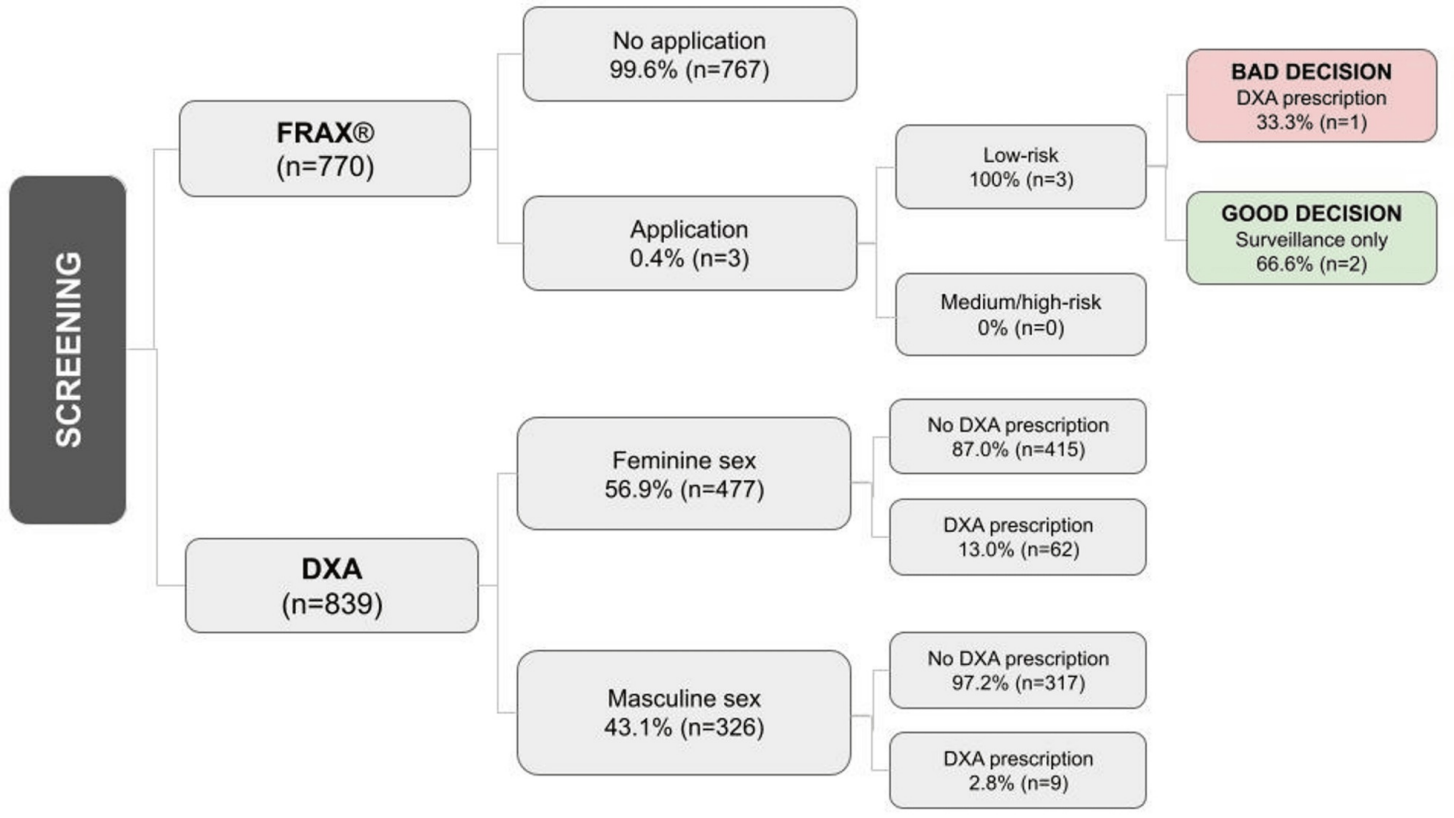
Osteoporosis screening cohort FRAX® application, management, and decision quality in the population aged 50 years and of DXA scan prescription in women aged 65 years and men aged 70 years. A good decision following a low-risk FRAX® assessment was defined as surveillance only, whereas a bad decision involved prescribing DXA or initiating pharmacological treatment. FRAX®: Fracture Risk Assessment Tool; DXA: dual X-ray absorptiometry scan

A visual representation of the screening cohort is presented in Figure 1.

OP-diagnosed cohort

Eight hundred and thirty-five (835) patients had a recorded diagnosis of OP between 2013 and 2023. After applying the exclusion criteria, 156 patients were excluded: nine due to death, 34 due to ongoing hospital-based follow-up, and 113 due to codification errors, namely misclassification of osteopenia as OP. The final OP cohort, therefore, comprised 679 patients. The median age of the participants was 71 years (mean 71.5 years), with a predominance of female subjects (90%, n=611).

Regarding OP duration, 82% of patients (n=557) had a diagnosis for more than two years. Within these, 49.2% (n=274) received a follow-up DXA prescription, although with a mean delay of 3.4 years. 

Concerning treatment, pharmacotherapy was initially prescribed for 88.4% of patients (n=600), mainly oral bisphosphonates (93%, n=558). Other prescriptions included strontium ranelate (2.7%, n=16), intravenous bisphosphonates and selective estrogen receptor modulators (1.8%, n=11, each), more than two different types of drugs at initiation (0.3%, n=2), and finally, denosumab and calcitonin (0.2%, n=1 each). Of note, 88.7% (n=602) out of the 679 patients with OP received oral bisphosphonates at some point during the study duration. 

Of the aforementioned 602 patients that received oral bisphosphonates, 6.6% (n=40) sustained fractures during treatment. The majority (90%, n=36) presented with one fracture, with the vertebrae being the most common site (36.1%, n=13), followed by the radius (22.2%, n=8), hip (16.7%, n=6), and other unspecified anatomical locations (25%, n=9). The remaining 10% (n=4) of fracture patients had multiple sites involved. Surprisingly, only one patient (n=1) was reported to have adverse gastrointestinal effects during treatment with oral bisphosphonates. No other adverse effects were reported. 

Furthermore, merely 29.4% (n=177) of patients who received oral bisphosphonates at any point adhered to the treatment regimen for a minimum of five years. Concurrently, a follow-up DXA scan after five years of this therapeutic approach was requested in 58.2% (n=103) of cases. Subsequent recorded femoral neck T-scores had a mean and median of -2.9 and -2.1, respectively. 

**Figure 2:**
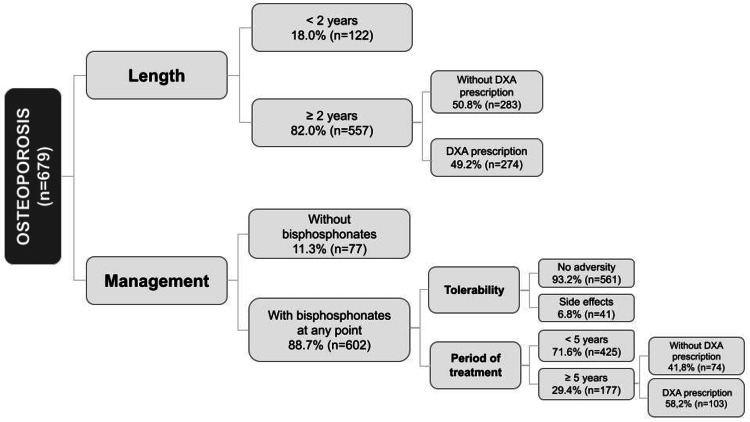
Frequencies regarding duration and management of osteoporosis in the disease cohort DXA: dual-energy X-ray absorptiometry scan

Further details on the duration and management of the OP cohort are exhibited in Figure 2.

Additionally, an analysis of adequate and inadequate bisphosphonate suspension showed that the majority of bisphosphonate suspensions were patient-initiated (48.2%, n=130). Notably, within the removals instigated by the PHC physician, the majority (61.5%, n=24) were deemed appropriate, in contrast to the patient-initiated discontinuations, where the majority (86.1%, n=112) did not exhibit apparent clinical criteria for suspension. Table 1 summarizes the reasons and appropriateness of bisphosphonate suspension decisions, providing insight into real-world clinical decision-making and its alignment with guideline-based recommendations.

**Table 1:** Bisphosphonate suspension in patients with osteoporosis Frequencies concerning bisphosphonate suspension decision in the osteoporosis cohort, specifically regarding the initiator of the decision, discontinuation criteria compliance, and decision appropriateness. Good and bad decisions were defined based on established drug holiday criteria and tolerability to bisphosphonates. Mixed decisions were defined based on the ambiguous management strategies applicable when a femoral neck T-score ≤–2.5 is observed after five years of bisphosphonate therapy. BMD: bone mineral density; DXA: dual-energy X-ray absorptiometry scan; OP: osteoporosis; DH: drug holiday

Bisphosphonate suspension in patients with OP				
Bisphosphonate suspension in OP patients	Without suspension (55.1%, n=332)		12.3% (n=41) of these cases met criteria for bisphosphonate suspension: 20 patients due to DH criteria (temporary suspension); 21 patients due to adverse effects (definitive suspension).	Bad decision
			4.5% (n=15) exhibited a femoral neck T score of ≤−2.5 after five years of bisphosphonate therapy, indicating a lack of improvement in BMD. Despite not meeting criteria for a DH or suspension due to side effects, these patients may warrant consideration for alternative OP therapy.	Mixed
			83.1% (n=276) of these cases did not meet criteria for bisphosphonate suspension: 221 patients exhibited tolerability but did not complete five years of bisphosphonate therapy; 48 patients did not undergo a DXA scan, and seven had no recorded T-scores, thereby precluding theoretical justification for therapy suspension.	Good decision
	Suspension (44.9%, n=270)	Medical suspension (14.4%, n=39)	28.2% (n=11) of these cases did not meet criteria for bisphosphonate suspension: Three patients exhibited tolerability but did not complete five years of bisphosphonate therapy; Five patients did not undergo a DXA scan, and three had no recorded T-scores, thereby precluding theoretical justification for therapy suspension.	Bad decision
			10.3% (n=4) exhibited a femoral neck T-score of ≤−2.5 after five years of bisphosphonate therapy, indicating a lack of improvement in BMD. Despite not meeting criteria for a DH or suspension due to side effects, these patients may warrant consideration for alternative OP therapy.	Mixed
			61.5% (n=24) of these cases met criteria for bisphosphonate suspension: 17 patients due to DH criteria (temporary suspension); Seven patients due to adverse effects (definitive suspension).	Good decision
		Patient voluntary suspension (48.2%, n=130)	86.1% (n=112) of these cases did not meet criteria for bisphosphonate suspension: 98 patients exhibited tolerability but did not complete five years of bisphosphonate therapy; 11 patients did not undergo a DXA scan, and three had no recorded T-scores, thereby precluding theoretical justification for therapy suspension.	Bad decision
			5.4% (n=7) exhibited a femoral neck T-score of ≤−2.5 after five years of bisphosphonate therapy, indicating a lack of improvement in BMD. Despite not meeting criteria for a DH or suspension due to side effects, these patients may warrant consideration for alternative OP therapy.	Mixed
			8.5% (n=11) of these cases did not meet criteria for bisphosphonate suspension: One patient due to DH criteria (temporary suspension); 10 patients due to adverse effects (definitive suspension).	Good decision
		Uncertain promoter (37.4%, n=101)	96.0% (n=97) of these cases did not meet criteria for bisphosphonate suspension: 80 patients exhibited tolerability but did not complete five years of bisphosphonate therapy; 15 patients did not undergo a DXA scan, and two had no recorded T-scores, thereby precluding theoretical justification for therapy suspension.	Bad decision
			4.0% (n=4) of these cases met criteria for bisphosphonate suspension: One patient due to DH criteria (temporary suspension); Three patients due to adverse effects (definitive suspension).	Good decision

Rheumatology Referrals

Of the total cohort of patients with OP (n=679), 16.9% (n=115) met criteria for referral to a rheumatology appointment. Of these, 75.7% (n=87) were not referred. The majority (58.6%, n=51) should have been referred due to bisphosphonate refractoriness (absence of improvement in follow-up DXA scans or fracture/intolerance in patients while on bisphosphonate therapy), followed by osteoporotic fractures at disease onset or during the course of the disease in patients who had never received bisphosphonates (33.3%, n=29), and finally due to suspected secondary OP (8.1%, n=7). Nevertheless, among the small proportion of patients referred to a hospital (4.1%, n=28), 100% did meet referral criteria, namely 50% (n=14) due to bisphosphonate refractoriness, 21.4% (n=6) due to osteoporotic fracture, and 28.6% (n=8) due to suspected secondary OP.

## Discussion

This study highlights significant deficiencies in OP screening and management within the PHC setting, spanning screening, follow-up, treatment adherence, and referral practices.

The near absence of FRAX® application in individuals aged exactly 50 years is particularly noteworthy, as this age represents a predefined preventive milestone in PHC. The negligible use of FRAX® suggests that OP risk stratification is not routinely considered during these consultations, representing a missed opportunity for early intervention. These findings are consistent with data showing low implementation rates of FRAX® in general practice in Portugal [13,14], despite endorsement by major guidelines [15-17]. This concordance with national data reinforces that the observed underutilization reflects a systemic pattern rather than isolated clinical behavior. Likely contributors include lack of integration into electronic medical records, limited physician familiarity, and competing priorities in time-constrained consultations.

Concurrently, the analysis of DXA prescription practices further underscores these screening shortcomings. Among women aged 65 years and men aged 70 years, the age thresholds for which DXA is recommended, a substantial proportion did not receive appropriate evaluation. This gap suggests that many individuals with low BMD remain undiagnosed, delaying preventive strategies that could significantly reduce fracture risk. Moreover, in a notable number of patients who had previously undergone DXA, no clear clinical justification was identified, pointing to a lack of standardized decision-making and, in some cases, misallocation of resources. Since the costs of diagnostic tests and procedures are monitored through FHU performance indicators, these pressures may influence clinical decisions, further highlighting the importance of aligning resource management with correct clinical practice. The discrepancy in DXA prescription by sex (more frequent in women, 13.2% vs. 3.4% in men) is expected given the stronger association with menopause, but the very low rates observed in eligible men reinforce the persistent under-recognition of OP in this population, who face substantial morbidity and mortality from osteoporotic fractures [18,19].

In the diagnosed OP population, the results suggest an initial proactive attitude from clinicians, with high rates of pharmacological intervention. Most patients received oral bisphosphonates, aligning with current recommendations that recognize these agents as first-line therapy due to their efficacy, safety, and cost-effectiveness [1,5,9].

However, treatment persistence and structured monitoring were clearly suboptimal. Fewer than one-third of patients maintained bisphosphonate therapy for five years, and follow-up DXA was frequently delayed or omitted altogether. This discrepancy between initiation and long-term adherence highlights challenges in sustained patient engagement and clinical follow-up, factors known to influence therapeutic success [20-22]. Almost half of patients missed a follow-up DXA, and when performed, the interval often exceeded the two to three years recommended by guidelines [5,9,16,23]. These findings are consistent with previous real-world studies reporting low long-term persistence with OP therapies and suggest that lack of structured follow-up may undermine effective risk reassessment. Consequently, risk stratification and treatment decisions, including continuation or suspension, were frequently undermined.

Therapeutic outcomes also reflected moderate efficacy: only 61.3% of patients with post-treatment DXA achieved a femoral neck T-score above −2.5. This aligns with established evidence of bisphosphonates’ capacity to reduce vertebral and non-vertebral fractures in the medium term [24,25], but underscores the need for structured reassessment and DH planning to balance benefits against risks of prolonged therapy. The frequent absence of key documentation, such as follow-up T-scores or explicit reasons for treatment suspension, further limits clinical decision-making, delays appropriate referral, and may contribute to inconsistent management.

Decisions regarding bisphosphonate suspension revealed additional inconsistencies. When initiated by physicians, most were appropriately justified, reflecting clinical judgment in line with recommendations [5,9,16,23]. In contrast, the overwhelming majority of patient-initiated suspensions lacked criteria, exposing communication gaps and insufficient patient education. These findings are closely linked to issues of tolerability and adherence: although bisphosphonates were generally well tolerated, with 93.2% of patients reporting no adverse effects, it is plausible that mild symptoms, such as gastro-oesophageal reflux or dyspepsia, were underreported, contributing to premature, undocumented discontinuations. Additional barriers to long-term treatment, including financial strain in polymedicated patients and low health literacy, have also been identified as key determinants of non-adherence in OP care [26,27]. Together, these factors highlight the importance of reinforcing shared decision-making, improving documentation of adverse effects, and clearly defining DH criteria in routine practice.

Finally, referral patterns shed light on systemic weaknesses in coordination between PHC and secondary care. Although only a minority of patients met criteria for referral, three-quarters of these were not referred, despite clear indications such as refractoriness or fragility fractures. Importantly, when referrals were made, all were appropriate, indicating that under-referral is more likely related to organizational or structural barriers than to clinical judgment. Contributing factors may include limited dissemination of referral criteria, lack of awareness of available hospital services, and overlapping responsibilities among specialties. Improving collaboration between PHC and hospital services, with simplified and transparent referral protocols, is essential to close this gap and ensure timely access to multidisciplinary care. These findings are in line with previous reports describing similar barriers in OP referral pathways, and several targeted strategies may be implemented to address the gaps identified in OP management across PHC settings, as represented in Table 2.

**Table 2:** Strategies for OP management Various targeted methods/approaches to bridge the gaps in OP management within the PHC setting in Portugal. FRAX®: Fracture Risk Assessment Tool; DXA: dual-energy X-ray absorptiometry; FHU: family health unit; OP: osteoporosis; PHC: primary healthcare

Strategy	Details
Notification for FRAX® score calculation	Including a notification in the “Alerts” section of the SClínico® platform could facilitate risk assessment during routine consultations, particularly at age 50, where it could be linked to other preventive interventions.
Integration of FRAX® port into the SClínico® platform	Adding a calculation tool in the individual patient record, similar to others already present, with guiding instructions based on the result.
Notification alert for DXA prescription	Development of automated prompts and alerts for DXA referrals at guideline-recommended ages could improve screening rates and standardise decision-making.
Establishment of structured OP follow-up consultations (analogous to those in place for hypertension and diabetes mellitus)	These could be supported by the development of quality improvement initiatives within FHU and the implementation of standardised protocols for clinical management and documentation during patient encounters.
Inclusion of OP-related metrics in performance indicators for PHC	Recognising OP as a priority chronic condition within national health targets would incentivise systematic assessment, treatment, and follow-up.
Training and audit for family physicians	Continuous professional development, combined with periodic audits, could improve OP management.
Enhanced collaboration between rheumatology services and PHC, particularly in the context of recently integrated health local units.	This may include outreach visits by hospital specialists to the FHU, provision of training sessions, and dissemination of referral guidelines or existing manuals. Such measures would promote clarity in referral pathways and foster multidisciplinary coordination.
Distribution of educational materials to patients, including leaflets outlining the nature of OP, available treatment options, and relevant precautions such as administration guidelines.	Improving patient literacy may support adherence and empower informed self-management.
Public health campaigns	Raising awareness of OP as a preventable and manageable disease - including among men - may help reduce stigma, increase screening uptake, and empower patients to engage in their care.

Limitations

This study has several limitations that warrant consideration. Firstly, its scope was geographically restricted to five FHUs in a single Portuguese region, which may limit the generalizability of findings to other settings with different healthcare structures, population characteristics, or clinical practices. Secondly, the retrospective design is inherently subject to potential misclassification, incomplete documentation, and limited data granularity, especially regarding clinical justifications for diagnostic or therapeutic decisions. Thirdly, the exclusive reliance on electronic medical records and prescribing platforms may underestimate the actual application of tools such as FRAX® or the rationale behind DXA requests, particularly when these are calculated or discussed but not systematically recorded. Additionally, treatment adherence was inferred from prescription records, which do not necessarily reflect real-world patient compliance. Finally, the study did not assess fracture incidence, functional outcomes, or quality-of-life measures, which limits the ability to correlate healthcare processes with long-term patient outcomes.

## Conclusions

OP remains underdiagnosed and inconsistently managed within Portuguese PHCs. Screening tools are underused, follow-up monitoring is irregular, adherence to bisphosphonates is poor, and referral to rheumatology is often neglected. While family physicians play a central role in the diagnosis and management of OP, contributing to the reduction of fracture incidence and costs and improvement in quality of life, the absence of structured systems and patient-centered approaches undermines continuity of care. Enhancing collaboration between PHC and specialist services is equally crucial to ensure timely referral and multidisciplinary management.

This study contributes valuable insight into Portuguese PHC, but not without its limitations. Therefore, further research, ideally prospective and multicentric, is warranted to better capture real-time clinical decision-making and its impact on long-term outcomes.
